# A Phase I study of NLG919 for adult patients with recurrent advanced solid tumors

**DOI:** 10.1186/2051-1426-2-S3-P250

**Published:** 2014-11-06

**Authors:** Asha Nayak, Zhonglin Hao, Ramses Sadek, Nicholas Vahanian, W Jay Ramsey, Eugene Kennedy, Mario Mautino, Charles Link, Pamela Bourbo, Robin Dobbins, Kelly Adams, Allison Diamond, Lisa Marshall, David H Munn, John Janik, Samir N Khleif

**Affiliations:** 1 Georgia Regents University, Augusta, GA, USA; 2 NewLink Genetics, Ames, IA, USA; 3 Georgia Regents University Cancer Center, Augusta, GA, USA

## 

Clinical Trial Registration Number: NCT02048709

## Background

The enzyme Indoleamine 2,3-dioxygenase (IDO1) catalyzes the cleavage of L-tryptophan, resulting in the production of kynurenine. Tryptophan depletion and kynurenine metabolites enhance the number and function of Tregs (suppressive arm) and inhibit effector T cells (stimulatory arm), regulating acquired local and peripheral immune tolerance. In cancer, IDO can either be expressed directly by the tumor cells themselves, or induced indirectly in host antigen presenting cells by the tumor and its expression has been associated with a worse clinical outcome in a variety of cancers. Indoximod (1-methyl D-tryptophan), the first IDO pathway inhibitor to enter human clinical trials, is currently being tested in multiple Phase II clinical trials. We are here to report preliminary Phase I data with another promising IDO pathway inhibitor, NLG919, a potent direct enzymatic inhibitor of IDO. While Indoximod and NLG919 both target the same IDO pathway, preclinical data clearly demonstrates that these compounds have different mechanisms of action and are synergistic. In preclinical models, NLG919 showed dose-dependent activation and proliferation of effector T cells, producing dramatic regression of large established tumors. Additionally, NLG919 showed enhanced immune activation and tumor regression when combined with Indoximod in this system.

## Methods

In this Phase I trial six dose levels of NLG919 are proposed for evaluation: 50, 100, 200, 400, 600 and 800 mg orally q12 h for 21 of 28 days in repeating cycles. Patients with advanced solid tumors that have progressed following standard therapy are eligible unless prior Ipilimumab or other CTLA4 targeted therapy has been administered. A single patient was treated at the first dose level and patients have been enrolled on subsequent dose levels with a standard 3 + 3 escalation scheme. Pharmacokinetics are evaluated for 48 hours following the first dose with no intervening doses at the initiation of the first cycle, and also at the end of the treatment cycle, day 21.

To date, no dose limiting toxicities have been observed and detailed updated study results will be presented. The primary objectives of the study are to evaluate the safety and toxicity of NLG919 in patients with advanced solid tumors and to define the maximum tolerated dose and recommended dose for Phase II studies. Secondary objectives are to measure pharmacokinetics, pharmacodynamics and evaluate any potential antitumor responses.

## Consent

Written informed consent was obtained from the patient for publication of this abstract and any accompanying images. A copy of the written consent is available for review by the Editor of this journal.

